# Job Resignation in Nursing Homes During COVID-19: The Role of Employer Communication and Worker Preparedness

**DOI:** 10.1093/geroni/igab046.956

**Published:** 2021-12-17

**Authors:** Verena Cimarolli, Natasha Bryant, Robyn Stone, Francesca Falzarano

**Affiliations:** 1 LeadingAge, Washington, District of Columbia, United States; 2 LeadingAge LTSS Center @UMass Boston, Washington, District of Columbia, United States; 3 Weill Cornell Medicine, Douglaston, New York, United States

## Abstract

Although research on factors mitigating the negative impact of strain/stress experienced by nursing home (NH) workers during the pandemic is emerging, there is no research on how COVID-19-related work stress and employer supports influence NH workers decision to resign. The purpose of this study was to investigate if high quality communication related to COVID-19 by the employer – a form of job support - can mitigate the impact of work stress on NH employees (N=1,730) decision to resign by optimizing employees’ preparedness to care for residents with COVID-19. Guided by the Job-Demands-Control-Support Model and employing path analyses, results indicate that higher stress was associated with greater likelihood of resigning, which operated through the paths of communication quality and preparedness. While higher stress was associated with less optimal quality of communication, good quality of communication was associated with more optimal preparedness which was associated with reduced likelihood of leaving one’s job.

